# Investigation on the Effect of Dynamic Focus Feeding and Widening Path in Nanosecond Laser Drilling

**DOI:** 10.3390/mi16101081

**Published:** 2025-09-25

**Authors:** Jianke Di, Jian Li

**Affiliations:** 1 Suzhou Delphi Laser Co., Ltd., Suzhou 215026, China; 2 School of Materials Science and Engineering, Jiangsu University, Zhenjiang 212013, China

**Keywords:** laser drilling, through-hole, superalloys, trepan, helical, dynamic focus feeding

## Abstract

Laser trepan drilling and laser helical drilling are typical methods for fabrication of micro through-holes through scanning laser beam. In the drilling process, the subsequent laser pulse may be occluded by the edge and the sputter deposition at the edge of the previous drilled trench. Dynamic focus feeding and widening path can be employed to lessen the occlusion effect and both of them are always employed in laser helical drilling. However, Widening the trench needs to remove more volume of material and may bring certain negative effects such as lowering the recoil pressure as well as less splashing melt due to the limited constraint of trench wall. The effects of dynamic feeding the focal plane and widening the scanning path on the quality and efficiency in the nanosecond laser drilling process were investigated through laser drilling holes with diameter of 500 μm on a 300 μm thick GH4169 plate. Results show that dynamic focus feeding is beneficial in both drilling efficiency and drilling quality. Through laser helical drilling with dynamic focus feeding, micro through-hole can be fabricated in 5 s, and both smaller tilting angle of 0.073 rad and smaller heat-affected zone of 0.63 mm in radius can be obtained. Widening scanning path is helpful to perforating rapidly but leads to much more recast layer coating. the quality of the micro through-holes depends not only on the utilization efficiency of the laser energy, but also on high temperature spatter deposition, which is the source of the difference between different drilling strategies. Due to the low cost in equipment and the better hole quality, the laser drilling, especially laser helical drilling, has potential applications ranging from aerospace fields to normal fields such as the agricultural machinery industry.

## 1. Introduction

In many mechanical devices, micro through-holes are a typical structure that is broadly used to control fluids flow [1,2,3,4,5]. In some high-end applications, both the high-quality requirements of micro through-holes and the requirements for high-performance materials call for reasonable machining technology for micro through-holes [6,7,8]. In order to process such through-holes, some nontraditional machining technologies such as electrochemical machining (ECM), electrical discharge machining (EDM) [9,10] and three-dimensional printing [11] have been employed in the industry. However, in these methods, the material should be suitable to the corresponding technology, and the shape of the through-holes depends on the tool shape which in turn is affected by the process. In such a context, laser drilling becomes a promising process for micro through-holes fabrication due to its advantages of non-contact characteristics, non-wear characteristics, and non-material selection characteristics [12,13,14,15].

**Figure 1:**
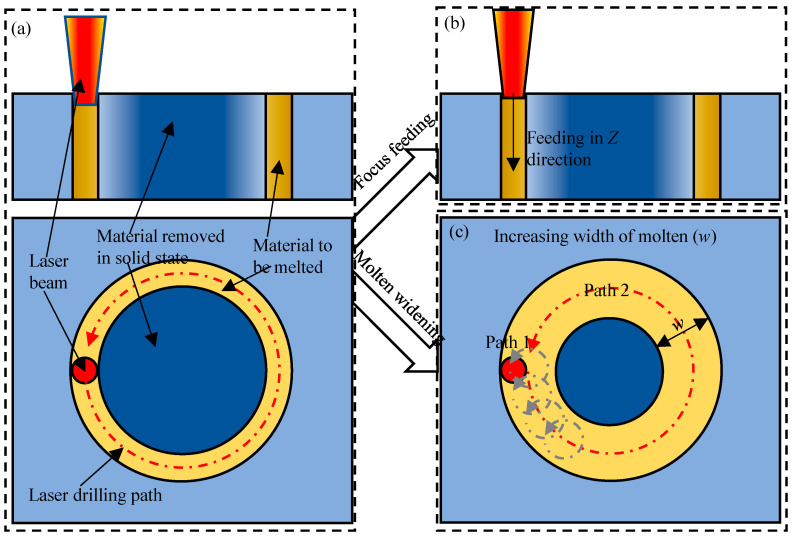
Schematic of the laser trepan drilling (**a**), laser helical drilling (**b**), and laser trepan drilling by widening the laser path (**c**).

**Figure 2:**
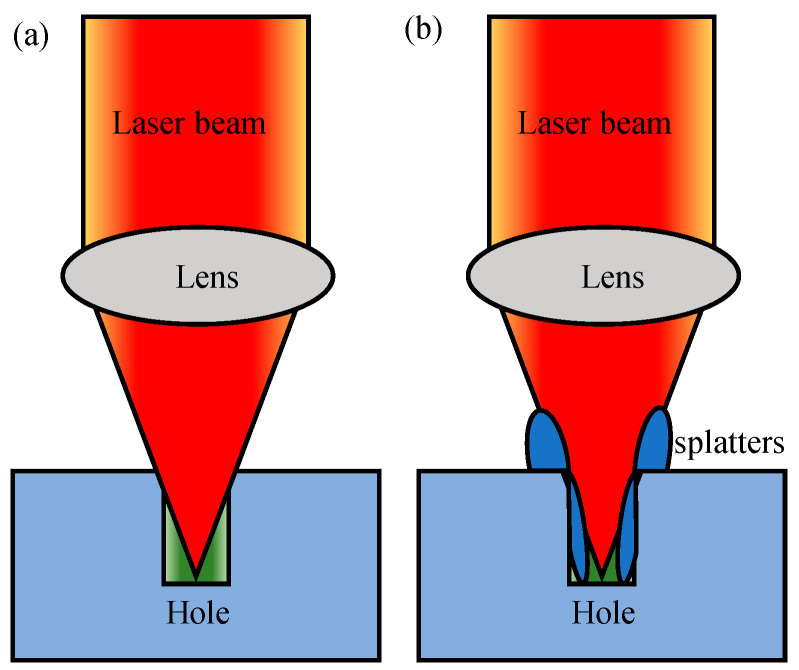
Schematic of the occlusion effect of trench edge (**a**) and the deposition of sputter to the edge of the trench (**b**).

At present, the single-pulse laser drilling [16,17], laser percussion drilling [18,19], laser trepan drilling [20,21] and laser helical drilling [22,23] have been developed. Once combined with femtosecond laser, these methods can be used to realize good hole shape and surface quality of hole wall [24,25,26]. Among these methods, laser trepan drilling [27,28] is an effective method to fabricate through-holes by melting less sputtering material, as shown in Figure 1. In laser trepan drilling, only the material within the path traveled by the laser is melted, and most of the material in the hole is removed in a solid form. Therefore, such a method is suitable to fabricate through-holes with larger diameter such as those in sprinkler [29,30,31] and can realize better inner wall surface quality. However, due to both the narrow scanning path and the fact that the laser focal plane remains unchanged during the machining process (the material to be melted cannot always keep in the beam focal plane), the efficiency of the successive processing stage is reduced [32,33], i.e., both the edge of the machined micro trench and the deposition of sputter to the edge of the trench reduces the laser energy access to the bottom of the trench (occlusion effect), as shown in Figure 2. This effect limits both the depth of the hole and the quality of the machined hole, which limits the application of laser trepan drilling in manufacturing through-holes such as nozzles of sprinklers [34,35,36]. Therefore, it is necessary to further explore and improve the process to improve the performance of the laser processing process.

To improve the efficiency of laser trepan drilling, one approach is to dynamically feed the position of the laser focal plane [37,38,39] (Figure 1b) at the right time, so that the material to be melted keeps at a high-intensity laser irradiation position. However, feeding the focal plane’s position requires adjusting the distance between the focusing lens and the sample, which should be realized synchronously with the scanning operation of the galvanometer. Recently, by placing the focusing lens on the front [40,41], it is possible to synchronously control both the focusing lens and the galvanometer scanner, i.e., laser helical drilling. The use of this system is expected to improve the efficiency of hole drilling. To relieve the occlusion effect of both the trench edge and the deposition, another way is to widen the width of the trench to be machined [42,43] (Figure 1c). Widening the trench can be achieved by scanning a series of overlapping circles or scanning a series of nested circles. To improve the efficiency of laser helical drilling, both feeding the focal plane and widening the trench were used in most former works [8,25] even drilling holes on thin samples [44]. Although widening the trench is helpful in avoiding occlusion effect, more volume of material needs to be removed. Moreover, widening the trench may bring certain negative effects in machining thinner plates, such as those in Ref. [44], e.g., lowering the recoil pressure as well as less splashing melt due to limited constraint of trench wall. In this paper, we will study the effect of dynamic focus feeding and widening trench on the laser trepan drilling in laser drilling micro through-holes on a GH4169 plate with thickness of 300 μm.

In laser drilling, although femtosecond laser can achieve better processing results [8,25,45,46,47,48,49], femtosecond laser is a typical processing with high investment, difficult maintenance, and low processing efficiency. As a low-cost equipment, nanosecond laser [50,51,52] and/or longer pulsed laser [53,54] even CW laser [55] is widely used in the industrial field and scientific research. However, the laser drilling with nanosecond laser is easy to cause serious recast layers and heat-affected zones especially in single-pulse and percussion drilling [56,57]. Such serious recast layers and heat-affected zones may limit the application of laser drilling in fabrication of some special nozzles [58,59,60]. If the recast layer and heat-affected zone can be effectively controlled, nanosecond laser will be more widely used in drilling through-holes on high-performance materials. For this reason, we investigate the effects of dynamic feeding the focal plane and widening the scanning path on the quality and efficiency in the nanosecond laser drilling process.

The nanosecond laser is used to drill holes on a 300 μm thick GH4169 plate. The micro through-holes are drilled by four processes, namely laser trepan drilling, laser helical drilling, laser trepan drilling with widening path and laser helical drilling with widening path. The diameter of the micro through-holes to be prepared is set at 500 μm. The deviation between the fabricated hole size and the ideal hole size, the hole morphology at the entrance end and the exit end, and the size of the heat-affected zone at the edge of the micro through-holes were analyzed.

## 2. Materials and Methods

The laser drilling experiments were carried out on GH4169 superalloy plate (the chemical composition is shown in Table 1) with dimensions of 20 mm × 20 mm × 0.3 mm. The workpiece was fixed on an *X*–*Y*–*Z* stage. The laser drilling experiments were performed using a Nd:YVO_4_ nanosecond laser at a wavelength of 532 nm and a pulse duration of 12 ns. The laser power, repetition rate, and scanning velocity were set as 20 W, 100 kHz, and 500 mm/s, respectively. The laser spot diameter is about 45 μm. As nanosecond laser drilling is a typical thermal process, the repetition rate of 100 kHz was chosen with reference to femtosecond laser drilling [8,25] although which may lead to certain thermal effect [61].

**Figure 3:**
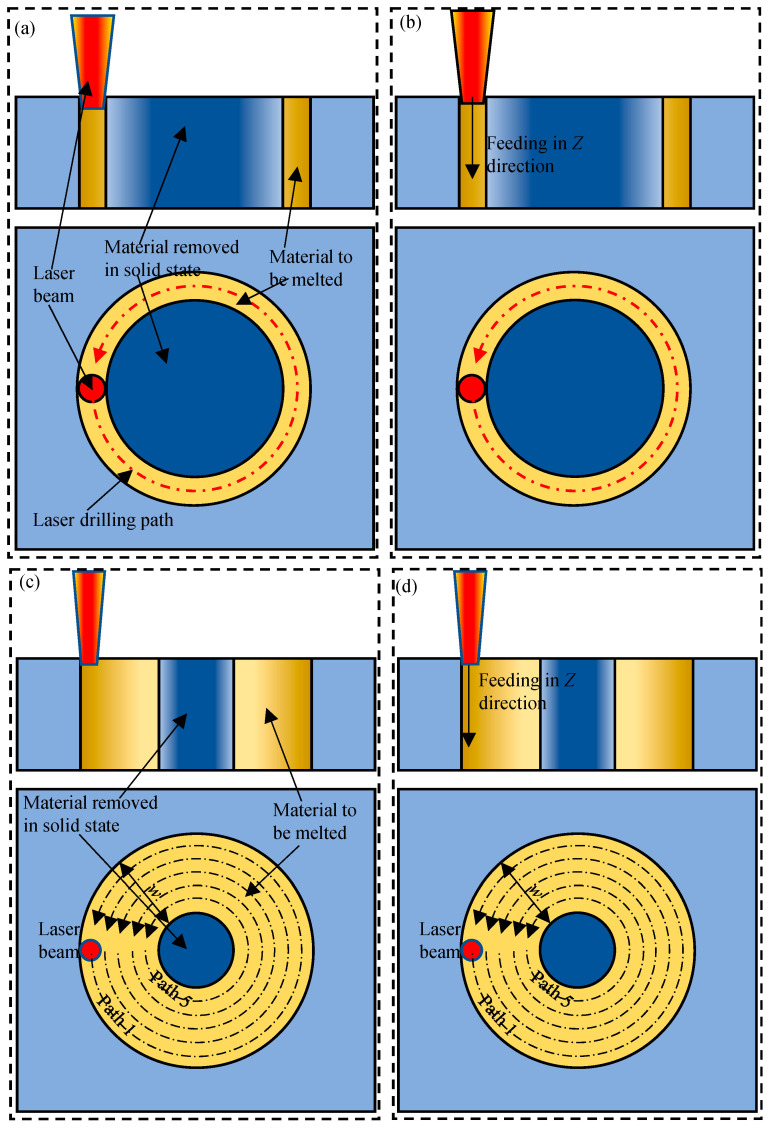
Schematic of (**a**) laser trepan drilling, (**b**) laser helical drilling, (**c**) laser trepan drilling with widening path, and (**d**) laser helical drilling with widening path.

Four types of laser drilling were investigated, i.e., laser trepan drilling, laser helical drilling, laser trepan drilling with widening path, and laser helical drilling with widening path, as schematic shown in Figure 3. In laser trepan drilling (Figure 3a), the laser beam moves along the outline of the micro-hole to be fabricated, and the material is removed through the scanning path to penetrate the workpiece. In the whole process, the focal plane of the laser beam remained the same. In laser helical drilling (Figure 3b), a 3D dynamic focusing system was used to realize reasonable dynamic focus along the *Z* axis. Combining the scanning operation and the dynamic focusing, the laser beam focus scans the material with a helical path. In laser trepan drilling with widening path (Figure 3c), the laser scans the material starting from the circular path 180 μm inward from the final outline of the micro-hole (i.e., *w* = 180 μm), while the focal plane of the laser beam remained the same. To widen the laser scanning path, five scanning paths with 45 μm spacing between paths were filled. Through this method, the laser is used to direct remove a 180 μm ring. In laser helical drilling with widening path (Figure 3d), the laser also scans the material starting from the circular path 180 μm inward from the final outline of the micro-hole and the focal plane is fed dynamically. In the drilling experiments, compressed air was used to blow away molten or vaporized material. All experiments were performed in air, and holes were drilled vertically at 90° to the workpiece.

After laser drilling, the morphology of both the entrance end and the exit end of the holes were examined by scanning electron microscopy (SEM) (SEM, FEI, Eindhoven, The Netherlands). The heat affected zone was investigated through a BX60M optical microscopy (Olympus, Tokyo, Japan).

## 3. Results

Four types of laser drilling were investigated by scanning electron microscopy. The parameters of the micro through-holes were measured from these SEM images. And the morphology on the SEM image were analyzed qualitatively.

### 3.1. Morphology of Micro Through-Holes Fabricated by Laser Drilling

#### 3.1.1. Laser Trepan Drilling

**Figure 4:**
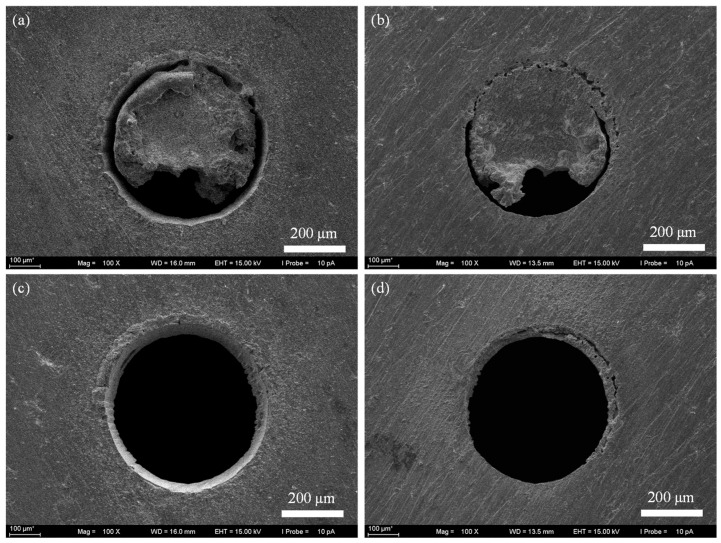
The SEM image of micro through-holes fabricated by laser trepan drilling. (**a**) The entrance end after drilling 5 s, (**b**) The exit end after drilling 5 s, (**c**) The entrance end after drilling 11 s, (**d**) The exit end after drilling 11 s.

Figure 4 shows the surface morphology at the entrance end and the exit end of the holes achieved by laser trepan drilling. It can be seen that after 5 s of processing, the solid material in the center of the micro holes did not fall off. And there was more material adhesion at the exit end of the micro holes (Figure 4b), making it difficult to remove the central material. After 11 s of processing (Figure 4c), the central material fell off, but sputters could be seen at the edge of the hole. And there was obviously material that had not been removed at the exit end of the hole, and the exit end of the hole was adhered to some recast layer.

#### 3.1.2. Laser Helical Drilling

**Figure 5:**
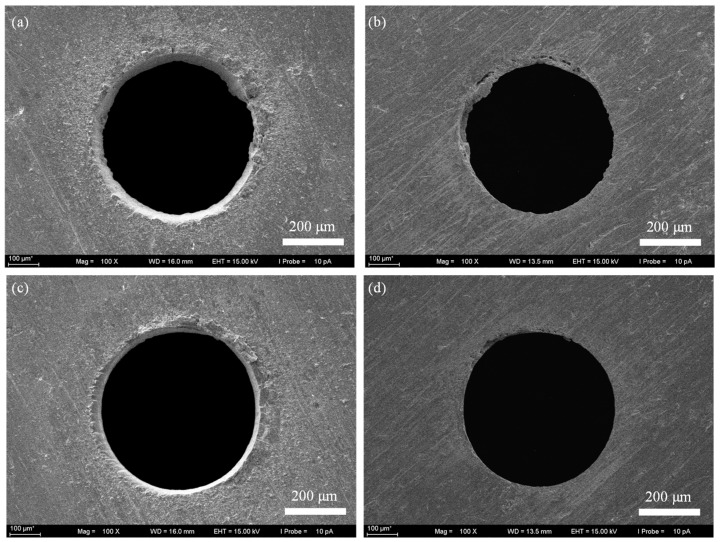
The SEM image of micro through-holes fabricated by laser helical drilling. (**a**) The entrance end after drilling 5 s, (**b**) The exit end after drilling 5 s, (**c**) The entrance end after drilling 12 s, (**d**) The exit end after drilling 12 s.

Figure 5 shows the micro hole morphology achieved by laser helical drilling. It can be seen that after 5 s of processing, the solid material in the center of the micro holes has been completely shed (Figure 5a), and only some material remains at the exit end of the micro holes (Figure 5b). After 12 s of processing (Figure 5c), the quality of the hole shape is improved, there is less sputter at the edge of the hole. There is no obvious material residue at the exit end of the micro hole (Figure 5d). The shape of the micro hole at the exit end is similar to that at the entrance end, indicating that the taper of the hole wall is effectively controlled. It can be seen that both the processing efficiency and quality of micro through-holes have been greatly improved by controlling the position of the laser beam focus (focusing lens position).

#### 3.1.3. Laser Trepan Drilling with Widening Path

**Figure 6:**
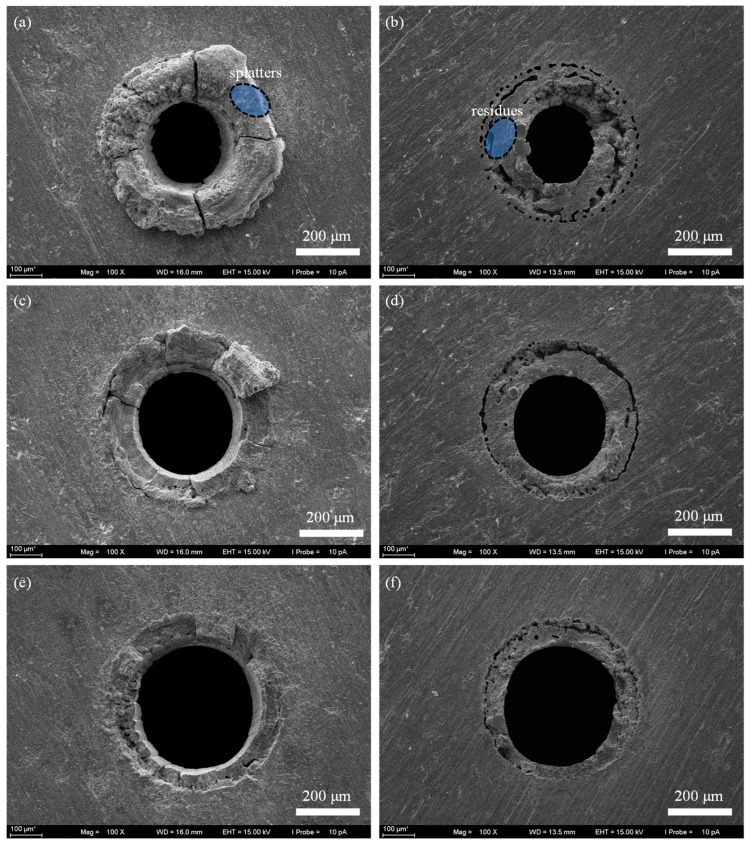
The SEM image of micro through-holes fabricated by laser trepan drilling with widening path. (**a**) The entrance end after drilling 3 s, (**b**) The exit end after drilling 3 s, (**c**) The entrance end after drilling 6 s, (**d**) The exit end after drilling 6 s, (**e**) The entrance end after drilling 15 s, (**f**) The exit end after drilling 15 s.

Figure 6 shows the micro holes morphology achieved by laser trepan drilling with widening path. It can be seen that for the 3 s machining, the solid material in the center of the micro holes has generally fallen off (Figure 6a), but there is a large amount of material residue at the entrance end and exit end of the micro holes (Figure 6a,b). For machining 6 s, the sputter of the holes was effectively removed (Figure 6c), and both the radius of the micro hole at the entrance end and the exit end increased (Figure 6c,d). After 15 s of processing (Figure 6e), the hole shape quality is improved, and the spatter at the edge of the micro hole is reduced, but the attachment of the recast layer is clearly visible at both the entrance end and the exit end of the micro hole (Figure 6e,f). These results indicate that increasing the width of the trench by controlling the scanning path of the laser beam does not achieve the ideal processing quality.

#### 3.1.4. Laser Helical Drilling with Widening Path

**Figure 7:**
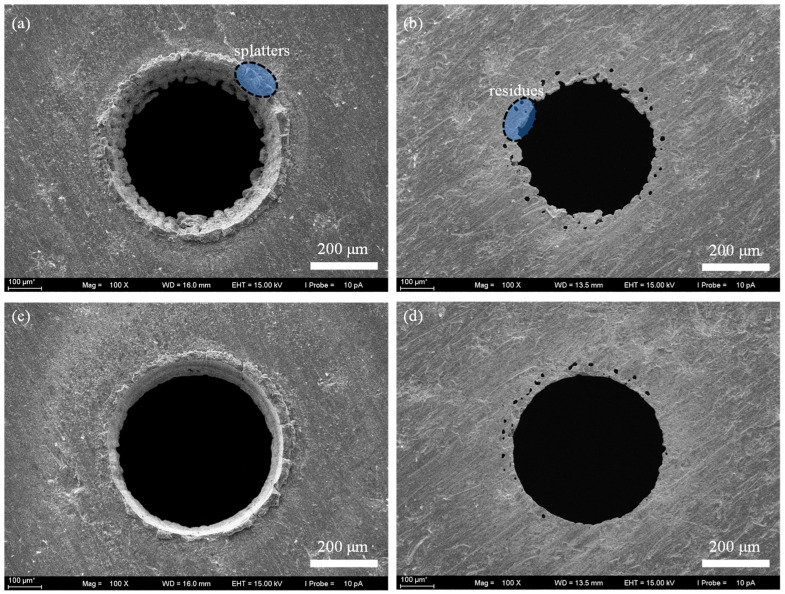
The SEM image of micro through-holes fabricated by laser helical drilling with widening path. (**a**) The entrance end after drilling 3 s, (**b**) The exit end after drilling 3 s, (**c**) The entrance end after drilling 8 s, (**d**) The exit end after drilling 8 s.

Figure 7 shows the micro holes morphology achieved by laser helical drilling with widening path. After 3 s laser drilling, the material at the center of the hole is effectively removed. However, there were more splatters left at the edge of the micro hole (Figure 7a,b), while the residue at the exit end of the hole was difficult to remove. Even after 8 s laser drilling, both the splatters and residue remain (Figure 7c,d). And some microscopic holes appeared at the edge (Figure 7b,d), indicating that laser helical drilling with widening path cannot achieve an ideal result.

### 3.2. Comparison of the Micro Hole Parameters in Different Laser Drilling

**Figure 8:**
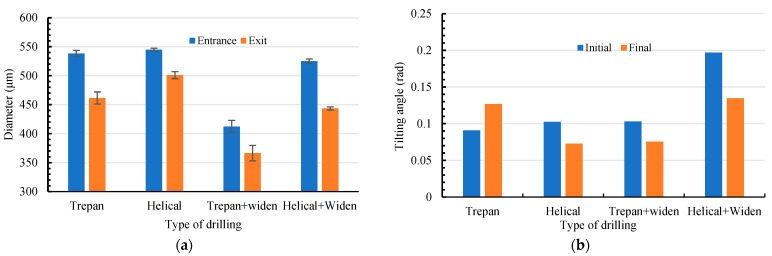
The parameters of micro holes at both the entrance end and the exit end in different laser drilling methods. (**a**) Diameter of micro hole at the entrance end and the exit end; (**b**) Tilting angle of the hole wall.

Figure 8a shows the diameter of micro holes at both the entrance end and the exit end in different laser drilling methods. The diameter of micro holes at both the entrance end in four types of laser drilling are, respectively, 538.2 ± 5.5 μm, 544.7 ± 2.7 μm, 412.2 ± 10.6 μm, and 524.9 ± 4.0 μm. Except that in laser helical drilling (544.7 ± 2.7 μm), the hole diameters especially in the case of widening the path are significantly different from the expected results (500 + 45 μm). The diameter of micro holes at both the entrance end in four types of laser drilling are, respectively, 461.6 ± 10.4 μm, 501.0 ± 6.1 μm, 366.5 ± 13.3 μm, and 443.6 ± 2.7 μm. It can be seen that the laser helical drilling brings the better results, i.e., the diameter of the micro holes at the entrance end and the exit end are close to the anticipated value of 500 + 45 μm and the difference between them is the less. Both the widening path experiments did not lead to reasonable results, which is even worse than that from the laser trepan drilling.

Figure 8b shows the tilting angle of the micro hole wall. The tilting angle *α* is calculated by *α* = arctan ((*D*_1_ − *D*_2_)/(2*h*)), where *D*_1_ and *D*_2_ are the diameter of the hole at the entrance end and the exit end, and *h* is the thickness of the sample [25]. In the initial stage, the tilting angles in four types of laser drilling are, respectively, 0.091 rad (only evaluated from the local profile of the scanning path), 0.103 rad, 0.103 rad, 0.197 rad. In the final stage, the tilting angles change to be 0.127 rad, 0.073 rad, 0.076 rad, 0.135 rad. It can be seen again that the best result is obtained from laser helical drilling, which is close to the value of holes fabricated by femtosecond laser drilling [8,25]. Although the laser trepan drilling with widening path realizes a letter tilting angle, one should keep in mind that such a small tilting angle is an illusion of the recast layer coated on the hole wall.

### 3.3. Comparison of the Heat Affected Zone in Different Laser Drilling

**Figure 9:**
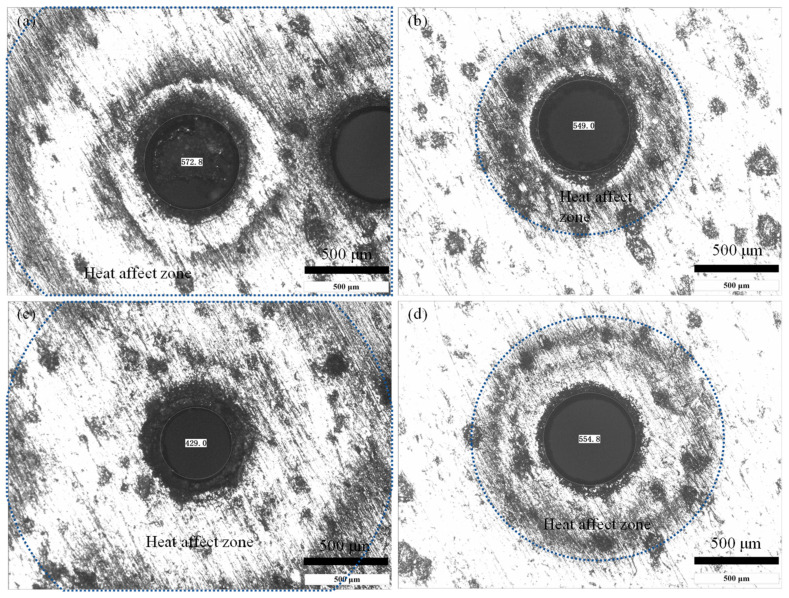
The heat affect zone in laser drilling. (**a**) Laser trepan drilling, (**b**) Laser helical drilling, (**c**) Laser trepan drilling with widening path, and (**d**) Laser helical drilling with widening path.

**Figure 10:**
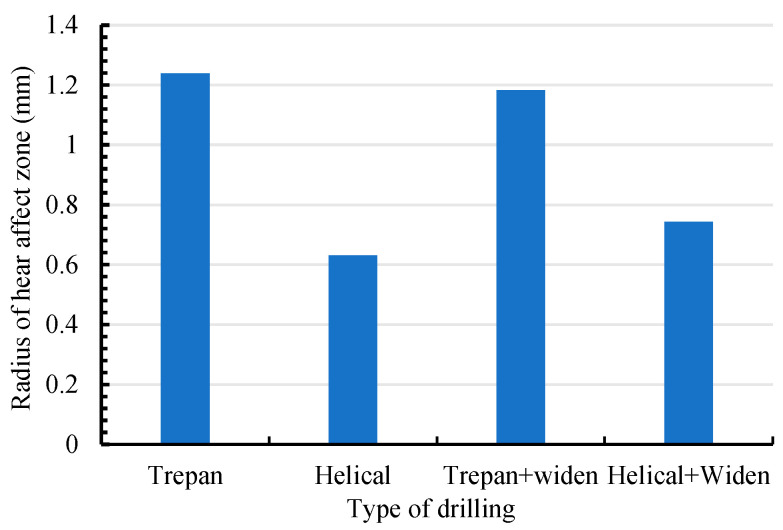
The radius of heat affect zone in laser drilling.

The method of laser drilling not only affects the quality of the hole and the residue of spatter but also affects the heat-affected zone due to the difference in the residue of the spatter and the heating process. Figure 9 and Figure 10 show the typical heat-affected zone size determined by different laser drilling methods.

It can be seen that for the case of laser trepan drilling (Figure 9a) the heat-affected zone is large (1.24 mm in radius). Because during most of the process, the material is not exactly in the laser beam focal plane, the utilization efficiency of the laser energy in material removal is low, and part of the laser energy propagates into the material near the ablated area. Moreover, poor evaporation and splashing conditions cause high temperature sputters to be deposited on the surface of the material, which in turn increases the heat-affected zone. For laser helical drilling (Figure 9b), due to the high intensity of the laser beam when it interacts with the material, the laser energy absorbed by the material is mainly used for splashing and evaporation, and less splash residue reduces the heat effect. Therefore, the heat-affected zone is the smallest in this situation (0.63 mm in radius). For laser trepan drilling with widening path (Figure 9c), even if the laser drilling time is short (3 s), the large residue of the melt makes the heat-affected zone larger (1.18 mm in radius, similar to that in laser trepan drilling), which also reflects the poor laser utilization efficiency in this case. Although the heat-affected zone can be improved by feeding the focal plane (Figure 9d), it (0.74 mm in radius) is still worse than that in laser helical drilling (Figure 9b).

## 4. Discussion

**Figure 11:**
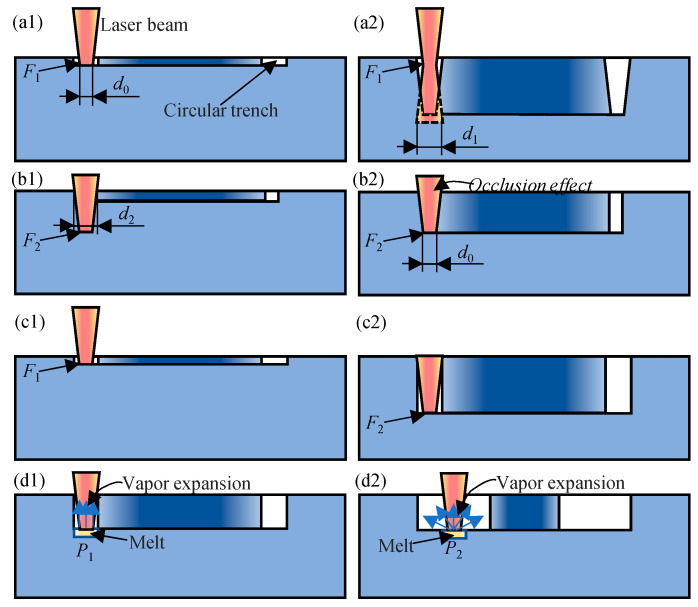
The schematic of laser–matter interaction in different scenarios. (**a1**,**a2**) Fixed laser focus near the surface, (**b1**,**b2**) fixed laser focus inside the material, (**c1**,**c2**) dynamically feeding the laser focus, and (**d1**,**d2**) comparison between the simple helical drilling and that with widening path.

From the above results, one can see that the best quality can be realized by dynamic feeding the laser beam focal plane, while widening the path contributes very little when drilling micro through-holes on a plate with thickness of 0.3 mm. In fact, dynamic feeding the focus is equivalent to widening the trench entrance to a certain degree. Importantly, the utilization efficiency of laser energy in melting and evaporation is increased by dynamic feeding the focus, which contributes to both effects of improving drilling efficiency and lessening heat affected zone. To clarify the effect of dynamic focus feeding and widening path, the mechanisms of laser–matter interaction in different situations are constructed, as shown in Figure 11.

In laser trepan drilling, due to the unchanging focal plane, the utilization efficiency of the laser energy is not very high unless the material to be ablated is exactly in the focal plane. If the focal plane is preset on the material surface (Figure 11a1), the intensity of the subsequent laser pulse is also less due to the defocus effect, i.e., the laser intensity decreases when the diameter of the laser beam at the plane of the material to be ablated increases from *d*_0_ to *d*_1_ (Figure 11a2). Therefore, the width of the trench decreases gradually. If the focal plane is preset inside the material (Figure 11b1), due to the less laser intensity (a wider laser beam) on the material surface, the entrance of the trench may be too narrow and most of the later laser pulses hardly reach the bottom of the trench due to the occlusion effect (Figure 11b2). Therefore, no matter where the laser beam focus is preset, the efficiency of laser trepan drilling is not very high and thus some recast layer adheres to the exit end of the hole, as shown in Figure 4.

In laser helical drilling process (Figure 11c1), if the material to be processed is in the focal plane (less out of focus), the laser intensity is the largest and thus the laser energy is effectively used for material melting and evaporation. The violent evaporation makes the melt splash more intense, resulting in both the wider entrance of the trench and a decrease in the sputter attached to the edge of the hole. Therefore, the occlusion effect of both the trench edge and the sputter adhered to the edge reduces. These two factors lead to a great improvement in the processing efficiency and quality of micro holes in laser helical drilling (Figure 11c2).

By scanning the laser beam in a wider area, although the width of the trench increased, the volume of melt removed by splashing and evaporation did not increase because the ablation time did not increase proportionally. And the increase in the laser ablation area led to a decrease in the amount of melt removed per unit area. At the same time, although the increase in the trench reduces the occlusion effect of the trench edge on the incident beam, it weakens the laser splash effect due to loss of constraint of trench wall (Figure 11d1,d2). In the simple laser helical drilling, the vapor expanded under constraint of trench wall and thus causes higher recoil pressure (Figure 11d1), while the vapor expansion in a wider space in laser helical drilling with widening path leads to lower recoil pressure (Figure 11d2). In the latter case, the melt, especially under the action of surface tension, is difficult to remove. Therefore, even after 15 s processing, the wall of the micro hole is still coated with a ~50 μm recast layer (formed from melt film on the microhole walls as revealed by high-speed synchrotron x-ray imaging [62]), which limits the application of such micro through-holes (Figure 6). By dynamic feeding the focal plane, the intensity of the laser can be enhanced. However, the wider and asymmetric trench less the self-focus effect of the trench wall on the laser beam, so the laser energy used to remove the material at the exit end of the hole is less. And thus, there was certain residue at the edge of the micro hole (Figure 7). Although widening drilling path does not realize better affect, it is still helpful in perforating rapidly, which may also be used in drilling thick plate and deserves further investigation.

The heat affected zone depends not only on the utilization efficiency of the laser energy, but also on high temperature spatter deposition. Certain models may be constructed based on simulation and experimental works in the future. And the best strategy of the helical path needs further investigation.

In the aerospace field, with the high requirement of combustion temperature, there are high requirements for both materials with better heat resistance and cooling film strategy. This requires micro through-holes with high precision, better surface quality, thinner recasting layers, and small heat-affected zones [63,64,65]. The micro through-holes fabricated by nanosecond laser helical drilling may have applications in these fields.

Due to the low cost of nanosecond laser, the laser trepan drilling and laser helical drilling can also be applied to achieve through-hole preparation in some normal fields such as the agricultural machinery industry. In these field, to improve the performance of nozzle, some special strategies are under investigation [66,67,68]. And employing non-circular nozzles can help drop formation [69,70], and the laser drilling method may be used to replace EDM [71,72] to realize better non-circular nozzles with broad scale.

## 5. Conclusions

Four nanosecond laser drilling strategies, namely laser trepan drilling, laser helical drilling, laser trepan drilling with widening path, and laser helical drilling with widening path, are investigated by laser drilling holes on the 300 μm thick GH4169 plate. It is found that by dynamic feeding the laser focus the laser helical drilling realizes a better surface quality and more precise parameters. By dynamically feeding the laser focus, the diameter of the hole changes from 538.2 ± 5.5 μm in laser trepan drilling to 544.7 ± 2.7 μm in laser helical drilling, which is well controlled around the expected one (500 + 45 μm). And the tilting angle changes from 0.127 rad to 0.073 rad, which is close to that in femtosecond laser drilling. The heat-affected zone varies from 1.24 mm to 0.63 mm in radius. Conversely, in drilling thin plates, widening the scanning path plays a negative role such as lowering the recoil pressure as well as less splashing melt due to limited constraint of trench wall. The diameter, the tilting angle, and the heat-affected zone are, respectively, 524.9 ± 4.0 μm, 0.135 rad, and 0.74 mm in laser helical drilling with widening path. In laser drilling, the quality of the micro through-holes depends not only on the utilization efficiency of the laser energy, but also on high temperature spatter deposition, which is the source of the difference between different drilling strategies. Due to the low cost of equipment and the better hole quality, laser drilling, especially laser helical drilling, has potential applications ranging from the aerospace field to normal fields such as the agricultural machinery industry.

## Figures and Tables

**Table 1:** Chemical composition of GH4169.

Fe	Ni	Cr	Co	Mo	C	Ti	Mn	Nb	Al
Bal	50–55	17–21	1	3	0.08	1	0.35	5.25	0.48

## Data Availability

No data was used for the research described in the article.
